# Hydrostatic Pressure Regulates MicroRNA Expression Levels in Osteoarthritic Chondrocyte Cultures via the Wnt/β-Catenin Pathway

**DOI:** 10.3390/ijms18010133

**Published:** 2017-01-12

**Authors:** Sara Cheleschi, Anna De Palma, Alessandra Pecorelli, Nicola Antonio Pascarelli, Giuseppe Valacchi, Giuseppe Belmonte, Serafino Carta, Mauro Galeazzi, Antonella Fioravanti

**Affiliations:** 1 Department of Medicine, Surgery and Neuroscience, Rheumatology Unit, University of Siena, Policlinico Le Scotte, Viale Bracci 1, 53100 Siena, Italy; saracheleschi@hotmail.com (S.C.); annadepalma90@live.it (A.D.P.); pascarelli@unisi.it (N.A.P.); mauro.galeazzi@unisi.it (M.G.); 2 Department of Medical Biotechnologies, University of Siena, Policlinico Le Scotte, Viale Bracci 1, 53100 Siena, Italy; 3 Department of Life Sciences and Biotechnology, University of Ferrara, Via Borsari, 44121 Ferrara, Italy; ale.pecorelli@gmail.com (A.P.); giuseppe.valacchi@unife.it (G.V.); belmonte.giuseppe@tiscali.it (G.B.); 4 Child Neuropsychiatry Unit, University Hospital, AOUS, Viale Bracci, 53100 Siena, Italy; 5 Plants for Human Health Institute, Department of Animal Sciences, NC State University, NC Research Campus, 600 Laureate Way, Kannapolis, NC 2808, USA; 6 Department of Medicine, Surgery and Neurosciences, Orthopaedic Section, University of Siena, Policlinico Le Scotte, Viale Bracci 1, 53100 Siena, Italy; s.carta@ao-siena.toscana.it

**Keywords:** microRNA, chondrocyte, osteoarthritis, mechanical loading, hydrostatic pressure, Wnt/β-catenin

## Abstract

Mechanical loading and hydrostatic pressure (HP) regulate chondrocytes’ metabolism; however, how mechanical stimulation acts remain unclear. MicroRNAs (miRNAs) play an important role in cartilage homeostasis, mechanotransduction, and in the pathogenesis of osteoarthritis (OA). This study investigated the effects of a cyclic HP (1–5 MPa), in both normal and OA human chondrocytes, on the expression of *miR-27a/b*, *miR-140*, *miR-146a/b*, and *miR-365*, and of their target genes (*MMP-13*, *ADAMTS-5*, *IGFBP-5*, and *HDAC-4*). Furthermore, we assessed the possible involvement of Wnt/β-catenin pathway in response to HP. Chondrocytes were exposed to HP for 3h and the evaluations were performed immediately after pressurization, and following 12, 24, and 48 h. Total RNA was extracted and used for real-time PCR. β-catenin was detected by Western blotting analysis and immunofluorescence. In OA chondrocytes, HP induced a significant increase (*p* < 0.01) of the expression levels of *miR-27a/b*, *miR-140*, and *miR-146a*, and a significant reduction (*p* < 0.01) of *miR-365* at all analyzed time points. *MMP-13*, *ADAMTS-5*, and *HDAC-4* were significantly downregulated following HP, while no significant modification was found for *IGFBP-5*. β-catenin levels were significantly increased (*p* < 0.001) in OA chondrocytes at basal conditions and significantly reduced (*p* < 0.01) by HP. Pressurization did not cause any significant modification in normal cells. In conclusion, in OA chondrocytes, HP restores the expression levels of some miRNAs, downregulates MMP-13, ADAMTS-5, and HDAC-4, and modulates the Wnt/β-catenin pathway activation.

## 1. Introduction

Osteoarthritis (OA) is a chronic degenerative joint disease characterized by degradation of articular cartilage, synovial inflammation, thickening of subchondral bone, and formation of osteophytes [1].

The major feature of OA is the destruction of extracellular matrix components (ECM) due to the imbalance towards catabolic activities of cartilage-degrading enzymes, like matrix metalloproteinases (MMPs) and disintegrin and metalloproteinase with thrombospondin motif (ADAMTS) [2]. The pathogenesis of OA is complex and not fully understood; multiple factors are involved in the development and progression of disease, such as genetic components and hereditability, aging, and biomechanical stimuli [1].

Mechanical force plays a fundamental role in regulating cartilagemorphogenesis and maintenance.

It has been demonstrated that cycles of loading, at which articular cartilage is physiologically subjected, are fundamental to regulate metabolic activity of chondrocytes, while an injuring overload is responsible for their damages and death [3,4].

A particular mechanical stress that acts in vivo is hydrostatic pressure (HP). Several in vitro studies have demonstrated that HP works as a modulator of cartilage metabolism [5], influencing cytoskeleton proteins, proteoglycans, and collagen synthesis with a magnitude, duration, and frequency of loading-dependent manner [6,7,8].

Recent studies have underlined the key role of microRNAs (miRNAs) in regulating chondrocyte functions [9,10]. miRNAs are a class of non-coding RNA molecules of 22–25 nucleotides that carry out epigenetic silencing of gene expression by binding the3’-untranslatedregion (3’ UTR) within target messenger RNAs (mRNA), in association with the RNA-induced silencing complex (RISC). The result of this binding determines the degradation of target mRNA, if the base pairing is perfect, or the translational repression if the match is partial [11]. miRNAs are implicated in the control of several cellular processes as well as in the development of various disorders [12]. The comparison between OA and normal cartilage specimens showed different miRNAs expression profiles, highlighting their involvement in the pathogenesis of OA [13,14,15,16]. Furthermore, bioinformatics approaches and in vitro experiments demonstrated that dysregulated miRNAs in OA target several genes encoding extracellular matrix remodeling proteins and pro-inflammatory factors.

miR-27a and miR-27b seem to be among the main studied miRNAs involved in OA; recent evidence has been reported that their lower expression in OA cartilage in comparison to normal cartilage was accompanied bydysregulation of both *MMP-13* and insulin-like growth factor binding protein *(IGFBP)-5* expression levels [17,18].

miR-140 could be considered a regulator of chondrocyte differentiation, bone development, and cartilage homeostasis. It has been demonstrated that a reduced expression of this miRNA in OA cartilage influenced the expression levels of its target genes *MMP-13*, *ADAMTS-5*, and *IGFBP-5* [17,19,20].

Many studies suggest the involvement of levels of miR-146a and miR-146b in the development and progression of OA, even though their expression in OA cartilage and their specific function has not yet been fully understood [21,22].

miR-365 is also correlated with cartilage catabolic processes in OA: its upregulation in OA chondrocyte cultures is induced by mechanical overloading, through the inhibition of its target gene histone deacetylase *(HDAC)-4*. Furthermore, miR-365 is a mediator of inflammatory response [23].

The biochemical and molecular mechanisms by which mechanical stress influences transcriptional activities in chondrocytes remain unclear. It has been reported that the Wnt/β-catenin pathway is associated with mechanical force application in joints, and that this signaling is activated after mechanical damage to cartilage [24,25].The Wnt/β-catenin pathway exerts a pivotal role in cartilage homeostasis and in OA pathogenesis [24]; in addition, it seems to be involved in the regulation of miRNA expression [26].

The purpose of this study was to investigate the possible effect of cyclic hydrostatic pressure (HP) (1–5 MPa, 0.25 Hz) on *miR-27a/b*, *miR-140*, *miR-146a/b*, and *miR-365* expression levels, as well as on the mRNA levels of their target genes, *MMP-13*, *ADAMTS-5*, *IGFBP-5*, and *HDAC-4*, in human normal and OA chondrocyte cultures. Furthermore, we evaluated the possible activation of the Wnt/β-catenin signal pathway through Western blotting analysis and immunofluorescence in order to establish its involvement in response to HP.

## 2. Results

### 2.1. Cell Viability

MTT test was performed to evaluate the effect of HP on chondrocyte survival. The mechanical loading tested in the study did not affect cell viability, neither immediately after HP nor at the various considered time points, in OA and normal chondrocytes (data not shown).These data were confirmed by a trypan blue test (data not shown).

### 2.2. miRNAs and Target Genes Expression

**Figure 1:**
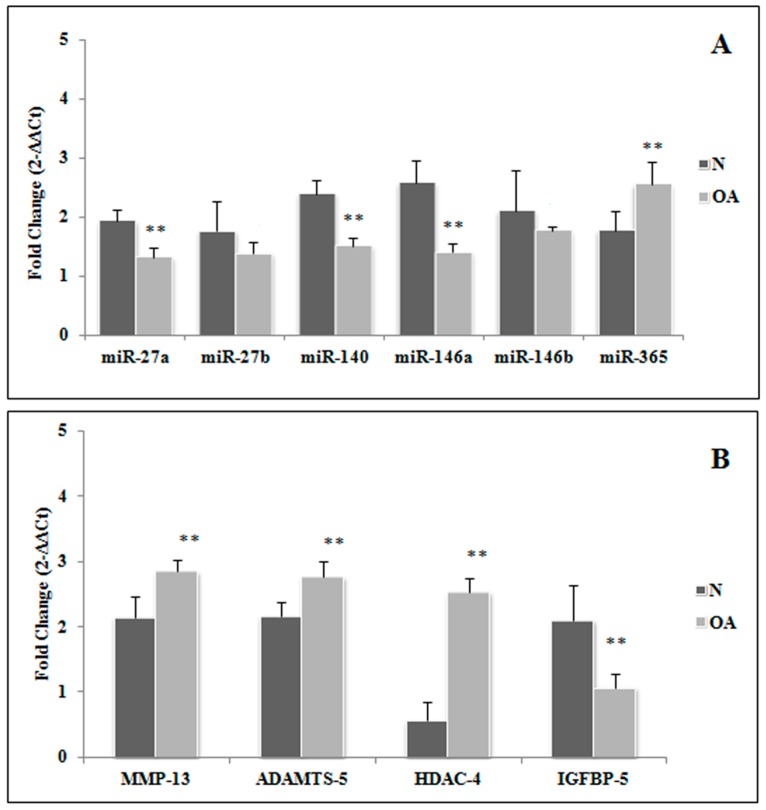
Expression levels of *miR-27a*, *miR-27b*, *miR-140*, *miR-146a*, *miR-146b*, and *miR-365* (**A**), and their target genes, *MMP-13*, *ADAMTS-5*, *HDAC4*, and *IGFBP-5* (**B**) in osteoarthritic (OA) and normal (N) chondrocytes at basal conditions. Data are expressed as mean ± standard deviation of triplicate values. ** *p* < 0.01. OA chondrocytes vs. normal chondrocytes. MMP-13: matrix metalloproteinase 13; ADAMTS-5:disintegrin and metalloproteinase with thrombospondin motif 5; HDAC-4 histone deacetylase 4; IGFBP-5: insulin-like growth factor binding protein 5.

At basal conditions we observed that the expression levels of *miR-27a*, *miR-140*, and *miR-146a* were significantly downregulated in OA cell cultures in comparison to normal (*p* < 0.01), while *miR-365* was upregulated in OA chondrocytes (*p* < 0.01). Concerning *miR-27b* and *miR-146b*, no significant differences between OA and normal cells were detected (Figure 1A).We also evaluated the transcriptional levels of these miRNA target genes. *MMP-13*, *ADAMTS-5*, and *HDAC-4* were found to be significantly increased in OA chondrocytes in comparison to normal cells at basal conditions (*p* < 0.01 for *MMP-13* and *ADAMTS-5*; *p* < 0.001 for *HDAC-4*). On the contrary, *IGFBP-5* gene expression appeared to be significantly downregulated in OA in comparison to normalchondrocytes, as evaluated at basal time (*p* < 0.01) (Figure 1B).

**Figure 2:**
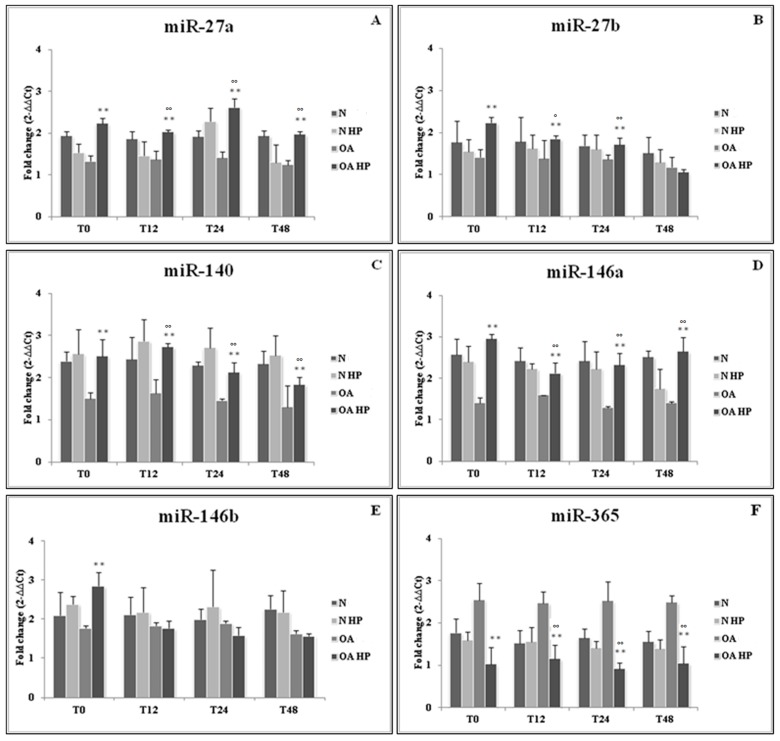
Effect of hydrostatic pressure (HP) exposure immediately after pressurization (T0), and after 12 (T12), 24 (T24), and 48 (T48) h, on the expression levels of *miR-27a* (**A**), *miR-27b* (**B**), *miR-140* (**C**), *miR-146a* (**D**), *miR-146b* (**E**), and *miR-365* (**F**) in normal (N) and osteoarthritic (OA) chondrocytes. N = normal cells with no HP exposure; N HP = normal cells subjected to HP; OA = OA cells with no HP exposure; OA HP = OA cells subjected to HP. Data were expressed as mean ± standard deviation of triplicate values. ** *p* < 0.01 OA HP vs. OA T0; ° *p* < 0.05, °° *p* < 0.01 OA HP vs. OA.

In addition, we evaluated the possible effect of HP on the same miRNAs and the related target genes expression. We detected a statistically significant up-regulation of *miR-27a/b*, *miR-140*, and *miR-146a/b* in OA chondrocytes (*p* < 0.01) immediately after pressurization (T0), compared to the unloaded controls. At different time points following HP application (T12, T24, and T48), high significant levels of *miR-27a*, *miR-140*, and *miR-146a* gene expression were maintained (*p* < 0.01) in comparison to the corresponding load-free conditions. The same results were obtained analyzing miR-27b levels, except for T48. The effect of loading on miR-146b increase was only restricted to the T0 time point. Concerning *miR-365*, our data showed its significant reduction after pressurization at the different analyzed time points in OA cells (*p* < 0.01). No significant modifications of all the analyzed miRNAs were observed over time in normal cells (Figure 2).

**Figure 3:**
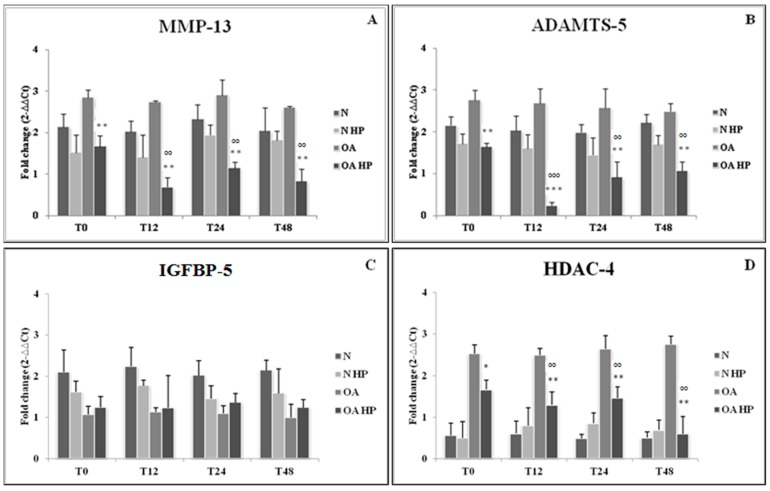
Effect of hydrostatic pressure (HP) exposure immediately after pressurization (T0), and after 12 (T12), 24 (T24), and 48 (T48) h, on the expression levels of target genes *MMP-13* (**A**), *ADAMTS-5* (**B**), *IGFBP-5* (**C**), and *HDAC-4* (**D**) in normal (N) and osteoarthritic (OA) chondrocytes. N = normal cells with no HP exposure; N HP = normal cells subjected to HP; OA = OA cells with no HP exposure; OA HP = OA cells subjected to HP. Data are expressed as mean ± standard deviation of triplicate values. * *p* < 0.05; ** *p* < 0.01; *** *p* < 0.001 OA HP vs. OA T0; °° *p* < 0.01, °°° *p* < 0.001 OA HP vs. OA. MMP-13: matrix metalloproteinase 13; ADAMTS-5: disintegrin and metalloproteinase with thrombospondin motif 5; IGFBP-5: insulin-like growth factor binding protein 5; HDAC-4 histone deacetylase 4.

Figure 3 shows a significant downregulation of *MMP-13*, *ADAMTS-5*, and *HDAC-4* expression levels immediately after loading (*p* < 0.01 for MMP-13 and ADAMTS-5; *p* < 0.05 for HDAC-4), as well as at the different analyzed time points compared to the respective unloaded controls (*p* < 0.01). On the contrary, *IGFBP-5* expression showed no significant modifications in response to HP. No significant changes in the evaluated target genes were observed over time in normal cells.

### 2.3. β-Catenin Protein Levels

**Figure 4:**
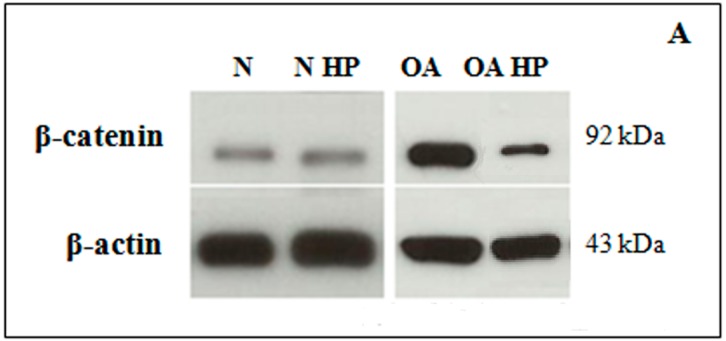

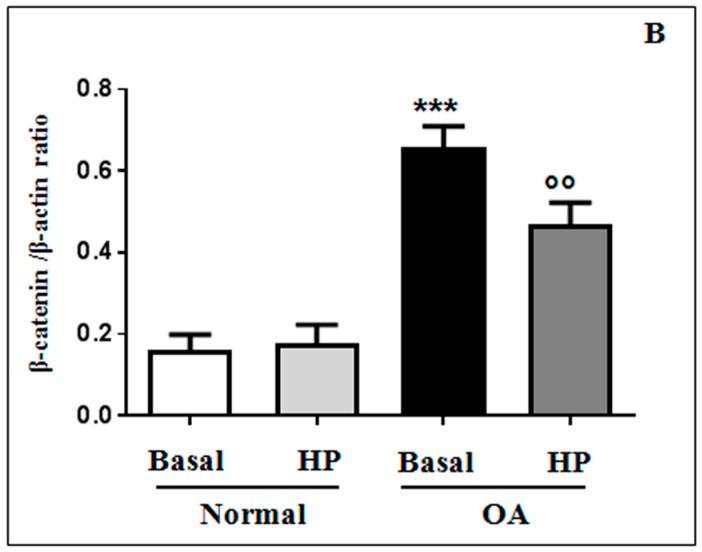
Westernblot of β-catenin. Representative immunoblotting images (**A**) and densitometric analysis (**B**) of β-catenin protein levels in human normal and OA chondrocytes evaluated at basal condition and immediately after the application of a HP (3 h). β-actin was used as an internal control. Data were expressed as mean ± standard deviation of three independent experiments. *** *p* < 0.001 OA chondrocytes vs. normal chondrocytes. °° *p* < 0.01 OA HP vs. OA basal conditions.

Western blot analysis of whole-cell lysates revealed clear bands at approximately 92 KDa for ß-catenin (Figure 4A). As reported in Figure 4B, densitometric quantification of the bands showed significantly higher (*p* < 0.001) β-catenin protein levels in OA samples, in comparison to normal, at basal conditions. Immediately after 3 h of HP exposure, a significant reduction of protein levels in OA chondrocytes was induced (*p* < 0.01), while no detectable differences were observed in normal cells following pressurization (Figure 4B). The loading control β-actin was not modulated by HP; in fact, it did not change significantly among the samples.

### 2.4. Immunofluorescence Analysis

**Figure 5:**
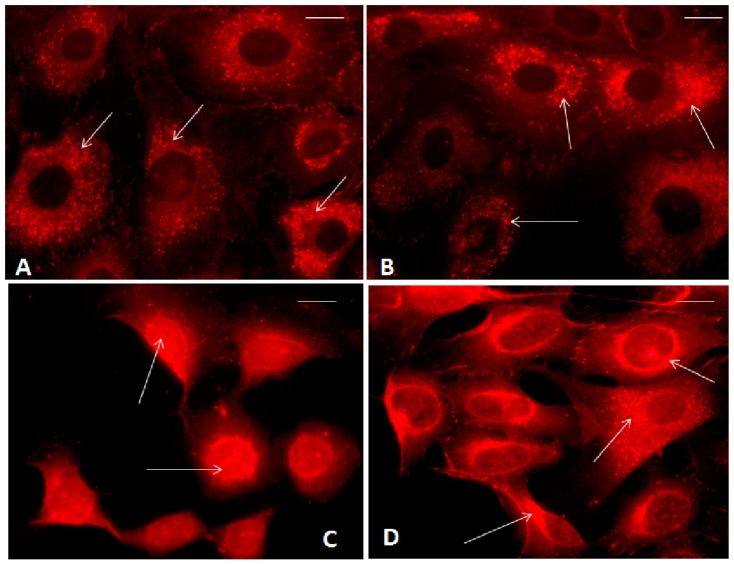
Immunofluorescence labeling of β-catenin localization in human normal and OA chondrocytes evaluated at basal conditions and immediately after the application of a HP (3 h). (**A**) Normal chondrocytes showed β-catenin cytoplasmic localization (**arrows**) at basal conditions; (**B**) Normal chondrocytes showed β-catenin cytoplasmic localization (**arrows**) after HP application; (**C**) OA chondrocytes showed nuclear β-catenin signal (**arrows**) at basal conditions; (**D**) OA chondrocytes showed reduced nuclear β-catenin signal and the presence of cytoplasmic localization (**arrows**) after HP application. Bar: 5 μm.

As reported in Figure 5, β-catenin signal was consistently positive in the cytoplasm and in the cell membranes of normal chondrocytes at basal conditions, while no evident positivity of the signal was shown in the nuclei (Figure 5A). The percentage of cells with β-catenin nuclear translocation was 12%, while the cytoplasmic percentage was 37%. No differences in its localization were observed after the application of our HP in normal chondrocytes (Figure 5B).

Figure 5C showed an abundant β-catenin nuclear translocation in OA chondrocytes evaluated at basal conditions; β-catenin nuclear positivity appeared to be reduced following 3 h of HP exposure; in this experimental condition a protein cytoplasmic positivity was detected (Figure 5D). The percentage of cells with β-catenin nuclear translocation at basal condition was 40% and after HP it was 15% (*p* < 0.01).

## 3. Discussion

The aim of this study was to evaluate if a cyclic HP could influence cell transcriptional activity, modifying *miR-27a/b*, *miR-140*, *miR-146a/b*, and *miR-365* expression levels, which are responsible for the regulation of mRNA levels of their target genes, *MMP-13*, *ADAMTS-5*, *IGFBP-5*, and *HDAC-4*, in normal and OA chondrocyte cultures.

One of the main factors regulating the metabolic activity of chondrocytes is the mechanical stimulus; articular cartilage is constantly subjected to loading cycles that depend on body weight, muscle tension, posture, and physical activity [27,28].

The typical composition and organization of cartilage extracellular matrix is maintained in a state of constant turnover by a balance of anabolic and catabolic activities of the chondrocytes, providing osmotic properties necessary for the ability of the tissue to resist compressive stresses [29].

Pressure levels on joints subjected to load have been measured in vivo; a pressure magnitude of 5 MPa is most often encountered in the knee joint during a normal gait [30]. In this study we tested the effects of HP with sinusoidal waves of intensity between 1 and 5 MPa, 0.25 Hz of frequency, in normal and OA chondrocyte cultures; this kind of loading, applied for a period of 3 h, approximately reproduces physiological conditions that occur in the human joints.

Our results showed that HP was able to modulate the expression levels of some miRNAs involved in the pathogenesis of the disease, in OA chondrocytes but not in normal cells. In fact the data confirmed previous evidence reporting that no changes occur when normal chondrocytes are subjected to pressure close to the physiological range of the human joint [27,31].

The role of miR-27a/b in OA has been pointed out; in fact, their gene expression was reduced in OA chondrocytes in comparison to normal [17,18], as confirmed by our data concerning *miR-27a* downregulation. There is no evidence about the possible effects of mechanical loading on modification of miR-27 a/b. Our study, for the first time, showed a positive effect of HP in OA cells, restoring the miRNA levels observed in normal conditions. This positive effect could be detected not only immediately after HP exposure, but was also maintained over time until 48 h.

Transfection of OA chondrocytes with anti-miRNA specific for miR-27a confirmed that *MMP-13* and *IGFBP-5* were its target genes, as predicted by bioinformatic approaches [17]. In addition, Akhtar et al. demonstrated an inverse correlation between expression levels of *miR-27b* and its target gene *MMP-13* in an in vitro study of human OA chondrocytes [18]. It is well known that MMPs play an important role in cartilage extracellular matrix degradation [32]; in particular, MMP-13 is one of the most common downstream targets, upregulated or inappropriately activated in OA by stress or pro-inflammatory signals [33].

IGFBP-5 is a protein that constitutes a reservoir of the anabolic factor IGF-1, promoting aggrecan and collagen fiber deposition and cell proliferation. Clemmons et al. showed that a downregulation of this protein in OA cartilage, associated with a reduced availability of IGF-1, contributed to extracellular matrix degradation [34], even though more recent evidence found an increase in both mRNA and protein IGFBP-5 synthesis in OA conditions [35].

As expected, our results reported a significant production of *MMP-13* in OA chondrocytes at basal conditions in comparison to normal cells; these expression levels were reduced following HP and were then maintained until 48 h after the application of mechanical loading. Interestingly, our data confirmed that IGFBP-5 mRNA levels were decreased in OA cells at baseline according to Tardif et al. [17]. However, the HP used in the present study induced a slight but not significant increase of IGFBP-5. Maybe it could be due to the involvement of different factors implicated in IGFBP-5 modulation [17].

IGFBP-5 3’-UTR is also targeted by miR-140, which plays an important role in OA pathogenesis; in fact, as shown by several studies on chondrocyte cultures, its expression was downregulated in injured cells, especially after pro-inflammatory cytokine stimulation [19,20]. The consequence of mechanical stress on *miR-140* regulation remained unknown. A significant increase of this miRNA level was induced by our pressurization system at all the analyzed time points, showing that miR-140 acts as a mechanical transduction modulator. The positive effect of *miR-140* upregulation is also accompanied by a significant decrease in the expression levels of *MMP-13* and *ADAMTS-5*, well known as matrix-degrading enzymes implicated in cartilage degradation during OA damage [36,37,38]. Transfection experiments with mimic miR-140 demonstrated the inhibition of MMP-13 and IGFBP-5, while in miR-140 knockout mice a significant increase of ADAMTS-5 was detected, demonstrating that *MMP-13*, *ADAMTS-5*, and *IGFBP-5* are miR-140 direct target genes [17,39].

In our study, the comparison between normal and OA chondrocytes at basal conditions, showed a significant downregulation of *miR-146a*, but not of *miR-146b*, in OA cells. Current knowledge about the expression trend of these miRNAs reported contrasting data. Li and colleagues affirmed that *miR-146a* is overexpressed in OA [22], while Jones et al. and Yamasaki et al. showed that *miR-146a* was intensely expressed in low-grade OA cartilage in comparison to normal tissue, but resulted progressively downregulated in the late stage of the disease, suggesting a potential role of this miRNA as a promoter of OA inflammation [13,21]. It was also demonstrated that a mechanical injuring pressure of 10 MPa induced the upregulation of *miR-146a* and a simultaneous activation of apoptosis in human normal chondrocyte cultures. Furthermore, Jin et al. observed, for the first time, that this miRNA plays a mechano-responsive activity, in a time-dependent manner [4]. In the present study the expression levels of *miR-146a* appeared to be upregulated following HP and this effect was maintained until 48 h. The obtained data seem to be in contrast with the current literature; however, this can be explained by the different experimental conditions applied, such as the cellular type analyzed and entity of loading, both for the intensity and for the duration. Recently, transfection experiments on human chondrocytes showed a negative regulation of *miR-146a* on *MMP-13* and *ADAMTS-5* levels, suggesting its anti-catabolic property [40]. Our data demonstrated a significant increase in *MMP-13* and *ADAMTS-5* in OA cultures at basal conditions and a significant reduction of their expression was obtained after the exposure of HP at all the analyzed time points.

There was no evidence regarding the evaluation of the expression levels of *miR-146b* neither in OA human chondrocyte culture nor after the application of mechanical loading. In the current experiment HP increased the expression levels of *miR-146b* only at the end of pressurization (T0); however, our experiment does not allow us to explain this result.

The present study confirms previous data reported by Yang et al. showing the overexpression of *miR-365* in OA chondrocyte cultures compared to normal cells. The upregulation of *miR-365* contributed to an abnormally accelerated differentiation and proliferation of hypertrophic chondrocytes and inflammatory cytokine production, increasing catabolic effects [23]. In addition, Yang et al. observed that *miR-365* was upregulated by an injuring mechanical loading, and by the stimulation with pro-inflammatory cytokines [23]. Following HP used in our study at all the analyzed time points, *miR-365* gene expression was significantly reduced in OA cultures.

HDAC-4 is a protein that plays a critical role in transcriptional regulation, cell cycle progression, and developmental events. It is a major regulator of cartilage development and endochondral ossification, functioning as a potent inhibitor of chondrocyte hypertrophy and differentiation [41]. Interestingly, the expression levels of this protein were higher in OA cartilage than in normal tissue [42], as also confirmed by our data. Furthermore, the inhibition of HDAC-4 by Vorinostat (SAHA) significantly reduced IL-1β/IL-6-induced expression of catabolic genes, especially *MMP-13*, in human OA cartilage explants and chondrocytes [43]. Following in vitro transfection studies, *HDAC-4* was identified as a direct target gene of *miR-365* [23].

Since the responsiveness of *miR-365* at mechanical loading, the *HDAC-4* expression levels modulation after pressurization was expected. In fact, our HP induced the *HDAC-4* down-regulation, decreasing the catabolic activities of chondrocytes associated to OA pathogenesis [44], even at 48 h following loading. The observed reduction of *miR-365* after HP, should be associated by an increase of HDAC-4 mRNA. However, Guan and coll. reported that miR-365 regulated the protein levels of HDAC-4, but not its mRNA levels [45]. Our evidence highlighted the beneficial role of HP suggesting the involvement of other factors activated by loading, different by miR-365, in the regulation of transcriptional activation of *HDAC-4*.

The canonical Wnt signaling pathway plays a fundamental role in embryonic skeletal development and articular cartilage growth and homeostasis [46,47,48]. The involvement of this pathway in the stimulation of cartilage matrix degradation in OA is well known. The activation of Wnt/β-catenin signaling in chondrocytes participates to cartilage matrix catabolism through MMPs and other proteases overexpression [49]. Our data appeared in agreement with the current literature. In fact, we showed a significant increase of total ß-catenin protein levels and its nuclear localization in OA chondrocytes in comparison to normal cells, confirming Wnt/β-catenin pathway activation; an increase of total β-catenin protein levels reveals an increase of the active non-phosphorylated form, since phospho-β-cateninis labelled to proteasomal degradation [50,51].

The important role of Wnt signaling in regulating miRNAs expression has been reported [26,52]. In addition, some in vitro and in vivo studies reported a link between the Wnt signaling pathway and mechanical loading [53,54]. In particular, Liu and colleagues demonstrated the activation of the Wnt/β-catenin pathway in an injured exercise-induced OA rat model [53], and Xu et al. showed the upregulation of β-catenin mRNA and protein expression in in vitro chondrocytes after intermittent cyclic mechanical tension [54]. In our study we evaluated β-catenin protein levels and their localization in cells to assess the possible involvement of this pathway in the modification of some miRNAs and their target gene expression levels, in normal and OA chondrocytes, following HP.

We analyzed β-catenin protein levels and cell localization after 3 h of HP, in agreement with a previous report suggesting that translocation of β-catenin is a rapid process [25]; in fact, Niu et al. observed the pathway activation following loading, with a plateau of 2 h. Our HP induced a significant reduction of β-catenin protein levels, as analyzed by Western blot in OA chondrocytes; these results are supported by immunofluorescence microscopy images showing the reduction of β-catenin inside the nuclei and the increase of its cytoplasmic localization after HP exposure. These preliminary results suggest a possible role of the Wnt/β-catenin pathway in mediating mechanical stimuli to regulate chondrocytes’ activities.

## 4. Materials and Methods

### 4.1. Cell Culture

Normal human articular cartilage was aseptically obtained from post-traumatic femoral heads of five subjects (three males and two females) with no known history of joint disease; macroscopic and microscopic analysis were performed to confirm that the tissue used was normal. OA human articular cartilage was obtained from the femoral heads of five patients (two males and three females) with hip OA defined by clinical and radiological American College of Rheumatology criteria [55], undergoing total hip replacement surgery; OA grade ranged from moderate to severe and cartilage showed typical osteoarthritic changes such as the presence of chondrocyte clusters, loss of metachromasia, and fibrillation (Mankin degree 3–7) [56]. OA chondrocytes originated from the area adjacent to the OA lesion. The femoral heads were provided by Orthopedic Surgery, University of Siena, Italy. The mean age of the group was 61 years (range: 49–71) for normal subjects and 70 years (range 63–74) for OA patients. The Ethics Committee of the Azienda Ospedaliera Universitaria Senese/Siena University Hospital approved the use of human articular specimens (decision no. 726/07) and patients signed to give informed consent.

Following surgery, the cartilage was aseptically dissected and minced into small pieces. The fragments were washed in DMEM with phenol red, containing 2% penicillin/streptomycin solution and 0.2% amphotericin B. The chondrocytes were isolated from the articular cartilage using sequential enzymatic digestion: 30 min with 0.1% hyaluronidase, 1 h with 0.5% pronase, and 1 h with 0.2% collagenase at 37 °C in the wash solution (DMEM + penicillin/streptomycin solution + amphotericin B). The resulting cell suspension was filtered twice using 70-μm nylon mesh, then washed and centrifuged for 5 min at 700× *g*. The trypan blue viability test pointed out a 90%–95% cell survival for both normal and OA chondrocytes. Cells were incubated for two weeks at 37 °C and 5% CO_2_ in DMEM culture medium containing 10% fetal calf serum, 200 U/mL penicillin, and 200 µg/mL streptomycin. The medium was changed three times per week. The cell morphology was examined daily with an inverted microscope (Olympus IMT-2, Tokyo, Japan) to avoid the dedifferentiation of expanded chondrocytes and preserve their phenotypic stability.

All experiments were performed by using three replicates of cell cultures from each single donor.

In the first passage normal and OA human chondrocytes were seeded in Petri dishes (35 × 10 mm) at a starting density of 1 × 10^5^ cells re-suspended in 2 mL of medium with phenol red containing 10% fetal calf serum, 200 U/mL penicillin, 200 U/mL streptomycin, and 2 mM glutamine until they became 85%–90% confluent. The primary single layer of chondrocyte cultures was evaluated before starting the experiment (basal conditions) and after the cyclic HP exposure.

### 4.2. Pressurization System

The pressure used for this study was generated by our prototype of pressurization system, provided by a hermetically sealed pressure chamber [57]. Petri dishes were filled with culture medium (2% fetal calf serum) and sealed with a special membrane (Surlyn 1801 Bynel CXA 3048 bilayer membrane, Du Pont) after excluding all air to avoid implosions due to the presence of air between the membrane and the medium. The dishes were placed inside a pressure chamber that was filled with distilled water and maintained at a constant temperature of 37 °C. The chondrocytes were then pressurized according to sinusoidal waves with a minimum pressure of 1 MPa, a maximum pressure of 5 MPa, and a frequency of 0.25 Hz for a period of 3 h.

Experiments have been performed using chondrocytes and supernatants collected at basal conditions and immediately after receiving pressure (T0), as well as after 12 h (T12), 24 h (T24), and 48 h (T48) of retention in culture, following pressurization. Some dishes, maintained in the same culture conditions for the same period of time without receiving any pressurization, were used as controls.

### 4.3. MTT Assay

Cell viability was evaluated at various time points named above by 3-(4,5-Dimethylthiazol-2-yl)-2,5-Diphenyltetrazolium Bromide MTT assay. Chondrocytes were in cubated for 3 h at 37 °C in a culture medium containing 10% of 5 mg/mL MTT (3-[4,4-dimethylthiazol-2-yl]-2,5-diphenyl-tetrazoliumbromide) (Sigma-Aldrich, Milan, Italy). The medium was then discarded and 0.2 mL of DMSO (Dimethyl sulfoxide, Rottapharm, Monza, Italy) was added to each well to solubilize the formazan crystals. The absorbance was measured at 570 nm using a microplate reader (BioTek Instruments, Inc., Winooski, VT, USA). Blank measurement was performed using a well without cells.

The percentage of cell survival was calculated as follows:

% Survival = (Absorbance of test)/(Absorbance of control) × 100(1)

The experiments were carried out on pre-confluent cell cultures to prevent contact inhibition influencing the results. Data were expressed as OD units per 10^4^ adherent cells.

### 4.4. RNA Extraction and RT-qPCR

Total RNA, including miRNA, was extracted using TriPure Isolation Reagent according to the manufacturer’s instructions(Roche Diagnostics GmbH, Mannheim, Germany) and was stored at −80 °C. The concentration, purity, and integrity of RNA were evaluated by measuring the OD at 260 nm and the 260/280 and 260/230 ratios by Nanodrop-1000 (Celbio, Milan, Italy). The quality of RNA was also checked through electrophoresis on agarose gel (FlashGel System, Lonza, Rockland, ME, USA). Reverse transcription for miRNA was performed using the cDNA miScript PCR Reverse Transcription (Qiagen, Hilde, Germany), while that for target genes was done using Quanti Tect Reverse Transcription Kit (Qiagen, Hilde, Germany), according to the manufacturer’s instructions.

MiRNAs and target genes were analyzed by real-time PCR using, respectively, miScript SYBR Green (Qiagen, Hilde, Germany) and Quanti Fast SYBR Green PCR (Qiagen) kits and the primers listed in Table 1. All qPCR reactions were performed in glass capillaries using a Light Cycler 1.0 (Roche Molecular Biochemicals, Mannheim, Germany) with Light Cycler Software Version 3.5. miRNA amplification was performed at 95 °C for 15′ for Hot Start polymerase activation, followed by 40 cycles of 15 s at 95 °C for denaturation, 30 s at 55 °C for annealing, and 30 s at 70 °C for elongation, according to the protocol. The reaction procedure for target genes amplification consisted of 5′ at 95 °C, 40cycles of 15 s at 95 °C, and 30 s at 60 °C. In the last step of both protocols, the temperature was raised from 60 to 95 °C at 0.1 °C/step to plot the melting curve.

To further analyze the dissociation curves, we visualized the amplicons’ length in an agarose gel to confirm the correct amplification of the resulting PCR products.

For data analysis, the *C*_t_ values in each sample and the efficiencies of the primer set were calculated using LinReg Software [58] and then converted into relative quantities (RQ) and normalized according to the Pfaffl model [59].

Normalization was carried out considering as housekeeping genes SNORD-25 for miRNAs, and ACTB for target genes. These genes were chosen, analyzing genes listed in Table 2, by software geNorm version 3.5 (Primer design, Southampton University’s School of Medicine, Southampton, UK), a popular algorithm to determine the most stable housekeeping gene from a set of tested candidate reference genes in a given sample panel. geNorm calculates the gene expression stability measure (M) for a reference gene as the average pairwise variation (V) for that gene with all other tested reference genes. Stepwise exclusion of the gene with the highest M value allows for ranking of tested genes according to their expression stability [60].

### 4.5. Western Blotting

Normal and OA chondrocytes at the first passage were seeded in Petri dishes (35 × 10 mm) at a starting density of 1 × 10^5^ cells/chamber and re-suspended in 2 mL of culture medium until they became 85%–90% confluent. Some dishes were subjected to 3 h of HP, while control dishes were maintained in the same culture conditions for the same period of time without receiving any pressurization. The samples were collected immediately after 3 h of HP to evaluate the β-catenin activation, in agreement with Niu et al. [56]. Total cell lysates were obtained with M-PER™ Mammalian Protein Extraction Reagent (Thermo-Fisher Scientific, Rockford, IL, USA) containing a protease inhibitor cocktail (Sigma-Aldrich S.r.l., Milan, Italy). Protein concentration was determined by the method of Bradford (Bio-Rad Laboratories S.r.l., Milan, Italy). Ten micrograms of proteins for each sample were loaded into 10% sodium dodecyl sulfate-polyacrylamide electrophoresis gels and separated by molecular size. Proteins were then transferred to a nitrocellulose membrane and, after blocking, incubated overnight at 4 °C with mouse monoclonal anti-total β-catenin (12F7) antibody (1:1000) (Santa Cruz Biotechnology, Santa Cruz, CA, USA). After incubation with secondary goat anti-mouse IgG (H+L)-HRP conjugate antibody (1:5000) (Bio-Rad Laboratories S.r.l.), the bound antibodies were detected using chemiluminescence (Bio-Rad Laboratories S.r.l.). The blots were then stripped and re-probed with HRP-conjugated β-actin (Sigma-Aldrich S.r.l.) as the loading control. Images of the bands were digitized and the densitometry of the bands was performed using Image-J software (LOCI, University of Wisconsin-Madison, Madison, WI, USA). Results were normalized with the respective loading control (β-actin).

### 4.6. Immunofluorescence

Normal and OA chondrocytes at the first passage were seeded on coverslips in Petri dishes (35 × 10 mm) at a starting scarce/low density of 8 × 10^4^ cells/chamber, to avoid possible cell overlapping, and re-suspended in 2 mL of culture medium until they became confluent. Some dishes were subjected to 3 h of HP, while others were used as controls. The cells were processed after 3 h to further confirm the pathway activation. The chondrocytes were rinsed twice in phosphate-buffered saline (PBS) and fixed in methanol for 20 min and in acetone for 5 min at −20 °C. The samples were then treated with a blocking solution (PBS, 1% bovine serum albumin (BSA), 5% normal goat serum (NGS)) for 20 min at room temperature and incubated overnight at 4 °C with mouse monoclonal anti-β-catenin (12F7) antibody (Santa Cruz Biotechnology) diluted 1:50 in PBS, 0.1% BSA, and 1% NGS. After three washes in PBS, the samples were treated with goat anti-mouse IgG-Texas Red conjugated antibody (Southern Biotechnology, Birmingham, AL, USA) diluted 1:100 in PBS, 0.1% BSA, and 1% NGS. Finally, the samples were washed three times in PBS and mounted with Vecta shield (Vector Labs, Burlingame, CA, USA). Negative controls were obtained by omitting the primary antibody. Observations were carried out with a Leitz Aristo plan fluorescence microscope. We randomly selected five visual fields for each sample and calculated the percentage of the number of cells exhibiting nuclear translocation of β-catenin to the total number of cells in each field of view [61]. A total of 100 normal and OA chondrocytes from each group were evaluated.

### 4.7. Statistical Analysis

The results were expressed as the mean ± standard deviation of triplicate values obtained from the experiments derived by each single donor. Data normal distribution was confirmed by Shapiro–Wilk, D’Agostino–Pearson, and Kolmogorov–Smirnov tests.

Real-time PCR were evaluated by one-way (ANOVA) with a Tukey’s post hoc test using 2^−ΔΔ*C*t^ values for each sample. Western blot data were analyzed by ANOVA with a Bonferroni posthoc test. Immunofluorescence staining was scored by the same researcher and the results were analyzed by chi-square test. All analyses were performed using the SAS System (SAS Institute Inc., Cary, NC, USA) and GraphPad Prism 6.1 (GraphPad Software, San Diego, CA, USA).A significant effect was indicated by a *p*-value <0.05.

## 5. Conclusions

Our results demonstrated for the first time the effect of a cyclic HP that approximates the physiological condition of human joints in restoring the expression levels of some miRNAs normally dysregulated in OA. Moreover, a significant reduction of transcriptional levels of the principal proteases responsible for OA cartilage matrix degradation was also detected. The positive influence of pressurization was observed immediately after loading and maintained over time, until 48 h. The observed transcriptional modifications of miRNAs and target genes in OA chondrocytes could be caused by HP-induced Wnt/β-catenin signaling pathway modulation.

These data suggest that physiological loading is able to influence the epigenetic regulation of chondrocyte homeostasis; this leads us to consider moderate physical exercise, in clinical practice, as a useful approach to limit articular cartilage damage in OA. Nevertheless, some study limitations need to be mentioned. Firstly, transfection experiments with specific anti-miRNA could be useful to confirm the regulation of target genes expression by the analyzed miRNAs. In addition, target genes’ protein levels should be detected to elucidate if transcriptional changes also reflect a translational regulation. Finally, experiments with specific Wnt/β-catenin inhibitors are recommended to confirm the role of this pathway in mediating the response to HP.

## Figures and Tables

**Table 1:** Primers for RT-qPCR.

**miRNA Gene**	**Cat. No. (Qiagen)**
*miR-27a*	MS00003241
*miR-27b*	MS00031668
*miR-140*	MS00003500
*miR-146a*	MS00003535
*miR-146b*	MS00003542
*miR-365*	MS00031801
**Target gene**	**Cat. No. (Qiagen)**
*MMP-13*	QT00001764
*IGFBP-5*	QT01450743
*HDAC-4*	QT00005810
*ADAMTS-5*	QT00011088

**Table 2:** Housekeeping candidate genes’ primers.

**Housekeeping for miRNAs**	**Cat. No. (Qiagen)**
*RNU1A*	MS00013986
*RNU5A*	MS00013993
*RNU6B*	MS00014000
*SCARNA-17*	MS00014014
*SNORA-73A*	MS00014021
*SNORD-25*	MS00014007
**Housekeeping for target genes**	**Cat. No. (Qiagen)**
*ACTB*	QT01680476
*HLA-G*	QT01839726
*HMBS*	QT00014462
